# Cost analysis of violence-related medical imaging in a Free State tertiary trauma unit

**DOI:** 10.4102/sajr.v23i1.1664

**Published:** 2019-01-08

**Authors:** Tiaan P. Steyn, Fekade A. Gebremariam

**Affiliations:** 1 Department of Clinical Imaging Sciences, Universitas Academic Hospital, University of the Free State, South Africa

## Abstract

**Background:**

Violence is a leading public health problem worldwide. Beyond the pain and suffering, violence has a significant economic impact on a country’s health, policing and judicial services. Because of the lack of current and comprehensive data in South Africa, local violence-related economic impact studies are largely estimations. Violence-related imaging expenditure, as a component of a public hospital’s expenditure, is yet to be determined.

**Objectives:**

The goals of this study were to measure the violence-related patient burden on Pelonomi Tertiary Hospital’s (PTH) trauma and radiology services, determine the imaging-component cost of violence-related injuries and calculate the financial burden violence has on the hospital’s expenditures.

**Method:**

From the PTH’s trauma unit patient registry, 1380 patients with violence-related injuries were consecutively sampled for 6 months ending 31 December 2017. Imaging investigations were documented and categorised according to the South African National Department of Health’s 2017 Uniform Patient Fee Schedule (UPFS). Descriptive analysis and cost calculations were performed using the 2017 UPFS tariff schedule and hospital-specific health efficiency indicators – patient-day equivalent and expenditure per patient-day equivalent.

**Results:**

Violence-related injuries accounted for 50.64% of all trauma department visits and received a total of 5475 imaging investigations. Violence-related imaging investigations represented 14.81% of all investigations performed by the radiology department in the study period. Overall violence-related admission costs amounted to R35 410 241.85 (8.33% of the hospital’s total expenditure), of which 20.08% (R7 108 845.00) was attributed to imaging investigations.

**Conclusion:**

Violence-related admissions had a high patient and financial burden on PTH. The pinnacle of healthcare cost saving is violence prevention; however, the cost-conscious radiologist could assist with cost saving if responsible and ethical imaging practices are followed.

## Introduction

In 2008, violence was earmarked as the leading cause of death (31.5%) among 31 177 unnatural deaths recorded in the National Injury Mortality Surveillance System report of South Africa. However, violence had already been declared a leading worldwide public health problem in 1996 at the Geneva World Health Assembly.^1^

Locally, the South African Police Service precincts in and around Bloemfontein reported a total of 8101 crimes in the categories of attempted murder, assault with the intent to inflict grievous bodily harm, common assault and robbery with aggravating circumstances. This was for the period 01 April 2017 to 31 March 2018.^2^

Beyond the pain and suffering caused, violence has a significant economic impact on society, both directly and indirectly. Some of the direct costs include those borne by the victim and perpetrator as a result of the violence, loss of productivity at work, government expenditure relating to healthcare, policing and judicial services.^3,4,5,6,7^

Because of a lack of comprehensive data in South Africa, local cost analysis studies can only estimate the economic impact of violence.^8^ The provincial Departments of Health showed the second highest expenditure of all government departments, spending close to R150 billion in the 2015–2016 fiscal year. This translated to an average cost of R3332 per person using public healthcare services in South Africa.^9,10,11^ This is an extremely high burden on both the national and provincial governments’ budgets when taking into consideration that, in 2014, healthcare contributed close to 9% of South Africa’s gross domestic product.^12^

Health information (e.g. cost analysis studies) is crucial in the planning, implementation, evaluation and management of healthcare resources, seeing that 82% of the South African population rely on the public healthcare system.^10,13,14,15,16^

There are limited studies in the South African literature that specifically focus on the cost of medical imaging in violence-related injuries. Imaging investigations are expensive and the South African Competition Commission’s Health Market Inquiry found that medical aid claims relating to imaging investigations increased by an average of 10.98% per year between 2011 and 2014.^17^

The objective of this study was twofold. Firstly, we aimed to determine the violence-related patient burden on trauma and radiology services at Pelonomi Tertiary Hospital (PTH), and secondly, to determine the cost of violence-related medical imaging and to contextualise this cost in terms of PTH’s total expenditure.

## Research methods and design

### Study design

This study was a descriptive cost analysis that aimed to measure the cost of violence-related medical imaging in the setting of a tertiary-level public hospital. Because of the complexity of cost analysis studies, the most practical method for estimating this cost was to use the South African National Department of Health’s 2017 Uniform Patient Fee Schedule (UPFS). The UPFS is a fee schedule used to bill patients using public healthcare facilities, and it is applicable to externally funded patients using public hospitals in all provinces throughout South Africa.

Therefore, the study could more accurately be defined as a descriptive cost analysis using the theoretical maximum cost that can be charged by the hospital for medical imaging. Although the data might not reflect the exact cost of performing the imaging investigations, it does provide an estimation thereof.

According to the UPFS, all imaging investigations are categorised from category A to category E according to the complexity of the investigation. Furthermore, each category consists of two fixed prices: a facility fee (depending on the service level of the hospital) and a professional fee (depending on the training level of the healthcare practitioner who interprets the imaging investigation). The 2017 UPFS imaging fees are presented in Table 1.^18^

**TABLE 1:** 2017 Uniform patient fee schedule imaging tariffs.

Category of investigation	Professional fee	Facility fee		
		Level 1	Level 2	Level 3^†^
**Category A**	**-**	**R72**	**R72**	**R80**
Allied health practitioner	R69	R141	R141	R149
General medical practitioner	R70	R142	R142	R150
Specialist medical practitioner	R131	R203	R203	R211^‡^
**Category B**	**-**	**R197**	**R197**	**R225**
Allied health practitioner	R184	R381	R381	R409
General medical practitioner	R189	R386	R386	R414
Specialist medical practitioner	R368	R565	R565	R593^‡^
**Category C**	**-**	**R456**	**R456**	**R521**
General medical practitioner	R294	R750	R750	R815
Specialist medical practitioner	R900	R1356	R1356	R1421^‡^
**Category D**	**-**	**R912**	**R912**	**R1041**
General medical practitioner	R585	R1497	R1497	R1626
Specialist medical practitioner	R1798	R2710	R2710	R2839^‡^
**Category E**	**-**	**R2324**	**R2324**	**R2657**
General medical practitioner	R2152	R4476	R4476	R4809
Specialist medical practitioner	R4488	R6812	R6812	R7145^‡^

† , Applicable to Pelonomi Tertiary Hospital;

‡ , Prices combining professional and facility fees applicable to this study.

### Research setting and sampling method

The PTH’s trauma unit served as the study population. This unit provides emergency medical care to the whole of central South Africa (Free State and parts of the Northern and Eastern Cape) and keeps a detailed electronic patient registry including the diagnosis and mechanisms of injury. Because of resource constraints and a high turnover of patients in the trauma unit, violence-related injuries for a 6-month period (01 July 2017 to 31 December 2017) were selectively used to ensure a manageable sample size.

Consecutive sampling was used to retrospectively select patients from the trauma unit’s patient registry. Inclusion criteria consisted of patients of any age who were attended to in the study period and who sustained violence-related injuries in the subgroups of ‘penetrating assaults’, ‘blunt assaults’, ‘combination of blunt and penetrating assaults’ or ‘gunshot injuries’.

Accidental and self-inflicted injuries were excluded, as well as patients who were dead on arrival. Patients who did not receive any imaging were excluded from cost calculations. Double registry entries and registry entries with missing data, which could not be recovered from the Hospital Information System, were also excluded.

### Data collection

The trauma unit’s electronic database (Microsoft Excel format) was filtered according to the inclusion and exclusion criteria. Each entry in the filtered database was manually cross referenced with the Hospital Information System (to eliminate database errors) and to obtain each patient’s discharge date. Hereafter, all directly identifiable patient information (name, surname, identity number) were removed from the database to ensure patient confidentiality. Patients were only identified with their hospital numbers.

Hospital numbers and admission dates were cross referenced with the Picture Archiving and Communication System (PACS). Each individual examination performed during the patient’s first admission was documented under the relevant modality and UPFS category A to E (using the UPFS procedure book). The procedure book contains more than 800 individual radiological investigations and procedures, and for this reason, investigations were not further subcategorised. Examinations were categorised from A to E and priced according to UPFS service level-3 facility fees and UPFS specialist professional fees.

The final database contained the number of imaging investigations performed for each patient categorised under the different imaging modalities and UPFS pricing categories.

### Data analysis

Descriptive statistics were calculated for continuous data and frequencies and percentages were calculated for categorical data.

Arithmetic and cost calculations, incorporating the 2017 UPFS tariffs, as well as healthcare efficiency indicators and relevant hospital expenses for the study period (obtained from the hospital’s information unit), were used to calculate relevant imaging costs and proportionality between imaging costs and hospital expenditure.

### Limitations

Imaging investigations performed outside the radiology department such as fluoroscopy in theatre and extended Focussed Assessment with Sonography in Trauma, done in the trauma unit, are not uploaded to the PACS and were subsequently not included in the study. This could have led to an underestimation in the total cost of imaging.

Contrast agents, administered during the imaging investigations were not included in the cost analysis because of varying cost between the different brands, as well as poor documentation of the exact amount and type of contrast that was administered.

Patients seen at PTH’s casualty department (a separately functioning department from the trauma unit) were not included in the study – this was because of the lack of an electronic patient registry.

## Ethical consideration

Ethical clearances were obtained from the University of the Free State’s Health Sciences Research Ethics Committee (UFS-HSD2018/0052), as well as the Free State Provincial Health Research Committee (FS_201803_010). All directly identifiable patient information (names, surnames, dates of birth, etc.) were removed to ensure patient confidentiality.

## Results

A total of 4966 patients were treated at PTH’s trauma unit in 2017, of which 2725 patients were treated during the period 01 July 2017 to 31 December 2017. Within this period, 1380 patients matched the inclusion and exclusion criteria. Violence-related injuries constituted 50.64% of all trauma department visits during the study period. Descriptive statistics are summarised in Table 2.

**TABLE 2:** Descriptive statistics.

Type of injury	*n*	%	Gender ratio (male:female)	Total patients (*n* = 1380)		Mean age in years (31.39)^‡^		Mean days spent in hospital	Total patients who received imaging (*n* = 1273)^§^
				Male	Female	Male	Female		
Blunt assaults	517	37.46^†^	7:1	450	67	32.48	34.11	12.07	485
Penetrating assaults	742	53.77^†^	9:1	670	72	30.40	31.26	7.45	679
Combination assaults	34	2.46^†^	16:1	32	2	29.06	26.50	9.18	31
Gunshot injuries	87	6.30^†^	7:1	76	11	31.93	34.73	10.71	78

† , Percentage of total injuries;

‡ , Mean age for entire sample;

§ , Totals used in cost analysis calculations.

The sample of 1380 patients included 1228 males (mean age of 31.22 years) and 152 females (mean age of 32.71 years). The study sample’s injuries consisted of 53.77% penetrating injuries, 37.46% blunt injuries, 2.46% combination injuries and 6.3% gunshot injuries. A combined 5475 individual imaging investigations were performed on 1273 patients in the study sample. A total of 107 patients did not receive any imaging and were excluded from further cost calculations.

General X-rays represented the bulk of the imaging investigations, totalling 3834 investigations, and amounted to R843 354.00. Computed tomography (CT) scans totalled 1566 investigations; however, they contributed to the highest cost of R5 957 280.00. A detailed breakdown of imaging cost per imaging modality is presented in Table 3. A total of 5475 imaging investigations amounted to R7 108 845.00, of which R2 631 939.00 represented level-3 hospital facility fees and R4 476 906.00 the specialist professional fees.

**TABLE 3:** Modality-specific facility and professional fees.

Fees	X-ray (*n* = 3834)	Ultrasound (*n* = 21)	CT (*n* = 1566)	MRI (*n* = 38)	Fluoroscopy (*n* = 16)	Total examinations (*n* = 5475)
Facility fees	R319 770.00	R5317.00	R2 197 422.00	R100 966.00	R8464.00	R2 631 939.00
Professional fees	R523 584.00	R8792.00	R3 759 858.00	R170 544.00	R14 128.00	R4 476 906.00

**Total**	**R843 354.00**	**R14 109.00**	**R5 957 280.00**	**R271 510.00**	**R22 592.00**	**R7 108 845.00**

CT, computed tomography; MRI, magnetic resonance imaging.

The average costs of imaging investigations for the different injury types are presented in Table 4.

**TABLE 4:** Average costs of imaging per injury type.

Injury type	Patients who received imaging (*n* = 1273)	Total imaging cost per injury type	Average imaging cost per injury type
Blunt assault	485	R3 259 685.00	R6721.00
Penetrating assault	679	R3 164 805.00	R4660.98
Combination assault	31	R228 742.00	R7378.77
Gunshot injury	78	R455 613.00	R5841.19

From the patient sample, 978 patients (70.87%) were admitted to hospital with a combined total of 9221 admission days. Patients who were not admitted to hospital totalled 295.

## Discussion

Although the UPFS applies to externally funded patients being treated in a public hospital, it should be noted that all state patients are billed according to the UPFS tariffs. The UPFS tariffs applicable to imaging investigations are used by public hospitals to incorporate the cost of imaging into performance indicators, as well as being used for financial analyses and budgeting.

After the invoicing is done, a patient may qualify for a rebate. The percentage rebate is dependent on the income level of the patient and other factors. Rebates may be up to 100% in H0 classified patients (pensioners and formally unemployed patients) and 0% in H3 patients (therefore full paying patients). Where rebates apply, the rebates are covered by the hospital. Aspects such as actual patient invoicing, debt collection and the reconciliation thereof, are beyond the scope of the study. Instead, the study used the theoretical maximum that could be charged to patients for medical imaging received in a tertiary government hospital – in other words, no rebates were considered. Therefore, if all patients in the study sample were regarded as H0 classified patients, the total invoice for imaging services would have had to be covered by the hospital.

The billing total of imaging performed in the study sample was R7 108 845.00. This includes both the professional fees and the facility fees, as set out in the UPFS. This billing total is an underestimation of the true amount because of the previously stated limitations.

In the study sample, general X-rays were the most frequently performed investigation (*n* = 3834) and contributed 11.9% to the total imaging bill, whereas CT scans (*n* = 1566) contributed 83.8% (R5 957 280.00) to the total bill. The modality with the third highest cost was magnetic resonance imaging (MRI) (*n* = 38) totalling R271 510.00, followed by ultrasound (*n* = 21) and fluoroscopy (*n* = 16), which totalled R14 109.00 and R22 592.00, respectively. In perspective, for every R1.00 the hospital billed for imaging in violence-related injuries, R0.84 was for CT scans, R0.12 for X-rays and R0.04 for MRI. The costs of ultrasound and fluoroscopic investigations were negligible, because records pertaining to ultrasounds performed by trauma personnel and screening procedures performed in theatre were not available for analysis.

Medical imaging, as a component of in-hospital services and costs, can be put into perspective by determining the patient burden on radiology services, as well as what share it holds in the hospital’s total expenditure. The radiology department performed a total of 36 956 imaging investigations in the study period. Violence-related imaging investigations, referred from the trauma unit, constituted 14.81% of all these investigations; however, this is an underestimation because of the study limitations and considering that none of the casualty department’s violence-related imaging referrals were included in the study.

To put the imaging costs into perspective, comparison to the hospital’s overall expenditure is needed. This can be done by comparing the data to the hospital’s expenditure per patient-day equivalent (ExPDE). Expenditure per patient-day equivalent is widely used as a proxy for a hospital’s cost-effectiveness and is calculated by dividing the hospital’s total expenditure, for a specific period, by the hospital’s patient-day equivalent (PDE) for the same period. A single PDE is a unit that can represent one or more patients depending on the hospital resources the patient or patients consume in a 24-hour period. This is calculated by using all inpatient days, half of out-patient visits and a third of emergency department visits. The rationale behind the formula is that out-patient visits and emergency department visits are estimated to consume one-half and one-third, respectively, of the resources spent on a single 24-hour patient admission. Therefore, ExPDE represents the average cost per patient per 24-hour services rendered by the hospital.^19^ Before comparing the data to the hospital’s ExPDE, an ‘imaging expenditure per patient-day equivalent’ was calculated in accordance with the ExPDE formula. This provided an average imaging cost per PDE for the study sample.

**FIGURE 1:**
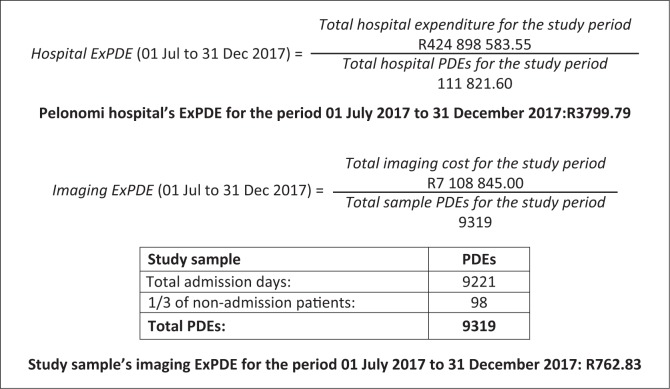
Hospital expenditure per patient day equivalent and imaging expenditure per patient day equivalent. PDE, patient-day equivalent; ExPDE, expenditure per patient-day equivalent.

The study sample included 978 patient hospital admissions with a total of 9221 admission days. This represented 9221 PDEs. One-third of the remaining non-admission patients represented 98 PDEs, which led to a total of 9319 PDEs in the study sample. Dividing the total imaging costs with the sample’s PDE, amounted to an imaging ExPDE of R762.83. Thus, the average cost of imaging per violence-related admission day was R762.83. Calculations are summarised in Figure 1.

Calculated from PTH’s financials for the study period, irrespective of the admitting department, the average cost per patient per 24-hours (ExPDE) was R3799.79. When the study sample was compared to the hospital’s ExPDE, violence-related imaging was found to constitute 20.08% of the hospital’s average cost per patient per 24-hour admission. Consequently, this proved that a considerable portion of money was spent on medical imaging in violence-related hospital admissions.

Using PTH’s ExPDE and the study sample’s PDEs, the hospital spent a total of R35 410 241.85 on violence-related trauma admissions for the last 6 months of 2017. This translated to 8.33% of the hospital’s total expenditure of R424 898 583.55 for this period. Seeing that violence is unlikely to disappear in the foreseeable future, all role players in the management chain of violence-related hospital admissions should be cost-conscious and avoid unnecessary expenditure.

Violence and its associated injuries are closely related; therefore, similar studies at set intervals may not only prove useful as cost analysis studies but also prove as useful indicators of the incidence of violence, the financial burden on healthcare services, as well as the effectiveness of existing violence prevention strategies and campaigns.

## Conclusion

Violence often leads to injury and the need for healthcare. During the last 6 months of 2017, 92.2% (*n* = 1273) of violence-related trauma department visits to PTH received imaging. The radiology department, therefore, plays an important role in the management chain of violence-related injuries. Violence-related admissions from the trauma unit cost PTH R35 410 241.85 in the last 6 months of 2017. An underestimated 20.08% of this expenditure was attributable to imaging investigations. Although we, as radiologists, can’t prevent violence at ground level, the services we provide constitute a significant portion of violence-related healthcare cost. In South Africa, with regular budget shortages, a recently announced technical recession and the anticipated implementation of National Health Insurance, the need for cost-saving strategies is becoming ever more important.
